# Importation of *Entamoeba histolytica* and predominance of *Klebsiella pneumoniae* in liver abscesses: a 7-year retrospective cohort study from the United Arab Emirates

**DOI:** 10.1186/s40794-021-00140-8

**Published:** 2021-06-12

**Authors:** Hussam Mousa, Ghada Salameh Mohammed Al-Bluwi, Zainab Fathi Mohammed Al Drini, Huda Imam Gasmelseed, Jamal Aldeen Alkoteesh, Zahir Osman Eltahir Babiker

**Affiliations:** 1 Department of Surgery, College of Medicine and Health Sciences, United Arab Emirates University, Al Ain, United Arab Emirates; 2 Surgical Institute, Al Ain Hospital, Al Ain, United Arab Emirates; 3 Department of Internal Medicine, College of Medicine and Health Sciences, United Arab Emirates University, Al Ain, United Arab Emirates; 4 Division of Infectious Diseases, Al Ain Hospital, Al Ain, United Arab Emirates; 5 Clinical Imaging Institute, Al Ain Hospital, Al Ain, United Arab Emirates; 6 Present Address: Division of Infectious Diseases, Sheikh Shakhbout Medical City in Partnership with Mayo Clinic, Abu Dhabi, United Arab Emirates

**Keywords:** Cohort studies, Amoebic liver abscess, Pyogenic liver abscess, *Klebsiella* infections, Treatment outcome, United Arab Emirates

## Abstract

**Background:**

There is a dearth of information on liver abscesses in the United Arab Emirates. Herein, we describe the clinical features of liver abscesses and determine their incidence rates and clinical outcomes.

**Methods:**

We retrospectively reviewed the clinical charts of adult patients with a primary diagnosis of liver abscess at a major hospital over a 7-year period.

**Results:**

Amongst 45 patients, 82.2% (37/45) had a pyogenic liver abscess (PLA) and 17.8% (8/45) had amoebic liver abscesses (ALA). Overall, patients were young (median age 42 years, IQR 35–52), mostly males (77.8%, 35/45) from the Indian subcontinent (55.6%, 25/45), presented with fever (88.9%, 40/45) and abdominal pain (88.9%, 40/45), and had a solitary abscess on imaging (71.1% (32/45). Crude annual incidence rates were 35.9/100,000 hospital admissions (95% CI 26.2–48.0) and 5.9/100,000 inhabitants (95% CI 4.3–7.9). All ALA patients were from the Indian subcontinent (100%, 8/8). *Klebsiella pneumoniae* was the most frequent pathogen in PLA (43.2% [16/37], 95% CI 27.1–60.5%). The hospital stay was shorter in ALA (7.5 days, IQR 7–8.5) than in PLA (14 days, IQR 9–17). No deaths were recorded within 30 days of hospitalisation.

**Conclusions:**

ALA was exclusively seen in migrants from the Indian subcontinent, suggesting importation. Further research to characterise *K. pneumoniae* isolates and assess potential risk factors is needed.

**Supplementary Information:**

The online version contains supplementary material available at 10.1186/s40794-021-00140-8.

## Introduction

Abscesses involving the liver represent serious intra-abdominal infections caused by bacteria, fungi, or parasites. Although no clear pathological mechanism for the development of pyogenic liver abscesses (PLA) can be established in some cases, they often arise due to biliary or portal foci of infection or following abdominal surgery [1–3]. Furthermore, hepatic artery chemoembolization or immunosuppressive therapy may predispose to infection of pre-existing hepatic lesions.

PLAs have historically been polymicrobial in nature, with *Escherichia coli* being reported as the main causative pathogen [4]. However, the rapid emergence and the spread of the hypervirulent strains of *Klebsiella pneumoniae* in Southeast Asia and further afield represent major public health threats. This is not only due to the serious metastatic complications associated with *K. pneumoniae* but also the development of antimicrobial resistance [3, 5]. On the other hand, an amoebic liver abscess (ALA) develops as a metastatic complication of intestinal infections due to *Entamoeba histolytica*. In high-income countries, ALAs are largely seen in migrants from endemic tropical regions or travellers returning from such regions [6].

There is a paucity of information on liver abscesses’ epidemiology in the Arabian Peninsula in general and the United Arab Emirates (UAE) in particular. In this study, we describe the demographic, clinical, radiological, and microbiological characteristics of patients with liver abscesses seen at a major UAE hospital. Given the ethnic diversity of the UAE’s population reflected in our region, we hypothesised that ALA might be imported rather than endemic and that *K. pneumoniae* might play an important role in PLA’s pathogenesis.

## Materials and methods

### Setting

Al Ain Hospital, a 402-bedded tertiary teaching hospital, is a designated regional infectious diseases centre serving an estimated population of 760,000 people living in the eastern region of the Emirate of Abu Dhabi in UAE [7].

### Study design

Electronic medical records were retrospectively searched for all adult patients (age > 15 years) who received a primary diagnosis of a liver abscess from 01 January 2012 through 31 December 2018.

### Case definitions

The definition of a liver abscess was based on a compatible clinical presentation, radiological findings consistent with abscess formation, and confirmation of pus or complete resolution of radiological abnormalities following antimicrobial therapy. Patients were treated empirically according to the local antimicrobial guidelines. Once microbiological confirmation and susceptibility profiles were available, targeted antimicrobial treatment was initiated.

### Diagnostic methods

Standard operating procedures were followed by the hospital laboratory for microbiological identification of organisms from blood or pus cultures. The presence of antibodies against *E. histolytica* was determined using an indirect haemagglutination assay (Cellognost Amoebiasis, Siemens Healthcare Diagnostics Products GmbH, Marburg, Germany).

### Data processing and analysis

Demographic, clinical, radiological, and microbiological data were collected and managed using REDCap electronic data capture tools hosted at the United Arab Emirates University’s portal [8, 9]. REDCap (Research Electronic Data Capture) is a secure, web-based software platform designed to support data capture for research studies, providing an intuitive interface for validated data capture; audit trails for tracking data manipulation and export procedures; automated export procedures for seamless data downloads to common statistical packages; and procedures for data integration and interoperability with external sources.

Data were inputted into Stata 15.1 (StataCorp, TX, USA) for statistical analysis. Continuous variables were summarized by means and standard deviations if normally distributed and by medians and interquartile ranges (IQRs) if not normally distributed. Fisher’s exact test was used to compare categorical variables. A *p-*value of < 0.05 was considered significant. Confidence intervals (CIs) were calculated with 95% precision.

Annual crude incidence rates for liver abscesses per 100,000 hospital admissions and inhabitants were calculated. All-cause outcomes were recorded as survived or died at 30 days. It was assumed that patients discharged before 30 days survived until 30 days.

Logistic regression was used to assess the association between PLA caused by *K. pneumoniae* and age, gender, region of ethnic origin, health insurance status, haemoglobin level, total white blood cell count, diabetes mellitus, malignancy, recent abdominal surgery, intensive care admission, and length of hospital stay. Crude and age-adjusted odds ratios (ORs) together with 95% CIs were calculated.

## Results

### Selection of patients

**Fig. 1:**
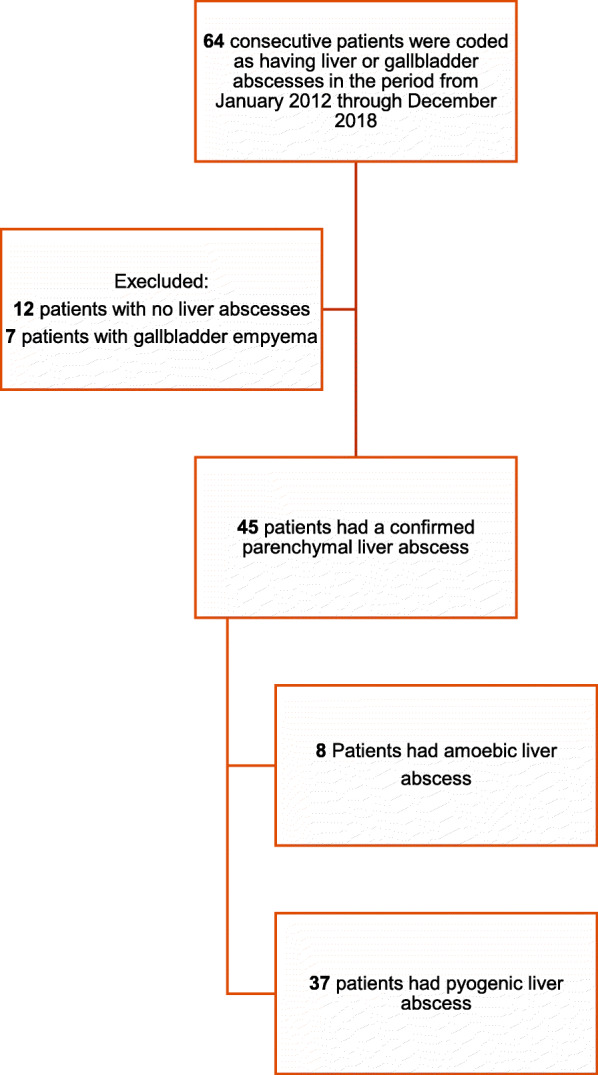
Flowchart showing selection of patients with liver abscesses included in the study

Of 125,356 hospital admissions during the study period, the medical records of 64 patients were initially screened for a potential primary diagnosis of liver abscess (Fig. 1). Seven patients with gallbladder empyema and further 12 patients without liver abscesses were excluded from the analysis. A total of 45 patients with confirmed liver abscesses were finally included in the study: eight with ALA (17.8%) and 37 with PLA (82.2%).

### Crude annual incidence rates

Overall, liver abscesses’ incidence rate was 35.9/100,000 hospital admissions/year (95% CI 26.2–48.0) and 5.9/100,000 inhabitants/year (95% 4.3–7.9). For ALA, the incidence rate was 6.4/100,000 hospital admissions/year (95% CI 2.8–12.6) and 1.1/100,000 inhabitants/year (95% CI 0.5–2.1). In contrast, PLA had an incidence rate of 29.5/100,000 hospital admissions/year (95% CI 20.8–40.7) and 4.9/100,000 inhabitants/year (95% CI 3.4–6.7).

### Socio-demographic and clinical features

Table 1 presents the socio-demographic and clinical features of patients with liver abscesses. Overall, the median age at presentation was 42 years (IQR 35–52). Most patients were males (77.8%, 35/45) with a male to female ratio of 3.5:1. None of the female patients (*n* 10) were pregnant. Most patients (55.6%, 25/45) were originally from the Indian subcontinent (11 from Bangladesh, nine from Pakistan, and five from India). Patients from the Indian subcontinent accounted for 100% (8/8) of those with ALA (five from Pakistan, two from India, and one from Bangladesh) and 45.9% (17/37) of those with PLA (10 from Bangladesh, four from Pakistan, and three from India). Most patients had health insurance coverage or self-pay status (77.8%, 35/45), while 22.2% (10/45) were eligible for free medical assistance.

**Table 1:** Socio-demographic and clinical features of patients presenting with liver abscesses, January 2012 through December 2018, Al Ain, United Arab Emirates

Characteristic	Amoebic liver abscess	Pyogenic liver abscess
Total number of patients *n* (%)	8 (17.8)	37 (82.2)
Age [years; median and interquartile range (IQR)]	39 (33.5–40.5)	46 (35–54)
Gender *n* (%)		
Male	7 (87.5)	28 (75.7)
Female	1 (12.5)	9 (24.3)
Region of ethnic origin *n* (%)		
Indian subcontinent	8 (100)	17 (45.9)
South East Asia	0 (0.0)	4 (10.8)
Middle East and North Africa	0 (0.0)	16 (43.2)
Health Insurance status *n* (%)		
Insured	6 (75)	26 (70.3)
Self-paying	1 (12.5)	2 (5.4)
Free medical assistance	1 (12.5)	9 (24.3)
Clinical presentation		
Fever *n* (%)	7 (87.5)	33 (89.2)
Abdominal pain *n* (%)	8 (100)	32 (86.5)
Diarrhoea *n* (%)	1 (12.5)	6 (16.2)
Vomiting *n* (%)	4 (50)	14 (37.8)
Jaundice *n* (%)	4 (50)	14 (37.8)
Weight loss *n* (%)	1 (12.5)	7 (18.9)
Temperature [degree Celsius, median (IQR)]	38.1 (37.5–38.8) [*n* 8]	37.9 (36.9–38.5) [*n* 35]
Systolic blood pressure [mmHg, median (IQR)]	136 (115.5–149) [*n* 8]	128 (117–139.5) [*n* 36]
Diastolic blood pressure [mmHg, median (IQR)]	77.5 (72–81.5) [*n* 8]	72 (65–80) [*n* 36]
Heart rate [median (IQR)]	104 (79.5–120) [*n* 8]	101 (89–119.5) [*n* 36]
Peripheral oxygen saturation [%, median (IQR)]	98 (96.5–99.5) [*n* 8]	98 (97–99) [*n* 35]
Respiratory rate [median (IQR)]	18 (18–18.5) [*n* 8]	18 (18–20) [*n* 36]
Modified early warning score	1.5 (1–3.5) [*n* 8]	2 (1–4) [*n* 35]
Potential risk factors *n* (%)		
Diabetes mellitus *n* (%)	0 (0.0)	12 (32.4)
Renal replacement therapy *n* (%)	0 (0.0)	1 (2.7)
Malignancy *n* (%)	0 (0)	3 (8.1)
Recent abdominal surgery *n* (%)	0 (0)	6 (16.2)
Recent foreign travel *n* (%)	2 (25)	2 (5.4)

Most patients with liver abscesses presented with fever (88.9%, 40/45) and abdominal pain (88.9%, 40/45). Other key symptoms were jaundice (40%, 18/45) and vomiting (40%, 18/45). On admission, patients with ALA and PLA had similar vital signs and modified early warning scores (MEWS).

In patients with PLA, 32.4% (12/37) had diabetes mellitus, 16.2% (6/37) had recent abdominal surgery, and 8.1% (3/37) had underlying malignancy. No patient with ALA had diabetes mellitus. Recent travel history was documented in 25% (2/8) of patients with ALA, and 5.4% (2/37) of patients with PLA. For patients with documented travel history, the median number of days since arrival from abroad was 70 (IQR 28–113). No patient with ALA or PLA had their HIV serostatus determined during their admission.

### Haematological and biochemical features

Overall, leucocytosis, hypoalbuminaemia, conjugated hyperbilirubinemia, and high serum gamma-glutamyl transferase were the most frequent abnormalities. There were no significant differences between patients with ALA and PLA regarding their haematological and biochemical characteristics (Supplementary Table 1).

### Microbiological features

All patients with ALA (100%, 8/8) were serologically confirmed and had no pathogens isolated from their cultures. Table 2 shows the microbiological features of patients with PLA. Overall, 97.3% (36/37) of patients with PLA had 42 liver pus cultures collected from them. Of these patients, 63.9% (23/36) had a monomicrobial infection, 11.1% (4/36) had a polymicrobial infection, and 25% (9/36) had no growth. Gram-negative bacilli were isolated from 66.7% (24/36) of these patients. On the other hand, 64.9% (24/37) of patients with PLA had blood cultures collected. All positive blood cultures (33.3%, 8/24) were monomicrobial, all of which were due to Gram-negative bacilli.

**Table 2:** Microbiological features of patients with pyogenic liver abscesses, January 2012 through December 2018, Al Ain, United Arab Emirates

Characteristic	Pyogenic liver abscess
Pus culture (*n* 42)^a^	
Gram-negative bacilli	
*Bacteroides species*	1
*Brucella species*	1
*Enterobacter cloacae*	1
*Escherichia coli*	2
*Klebsiella oxytoca*	1
*Klebsiella ozaenae*	1
*Klebsiella pneumoniae*	16
*Raoultella ornithinolytica*	1
Gram-positive cocci	
*Enterococcus faecalis*	1
*Staphylococcus aureus*	1
Viridans group streptococci	7
No growth	9
Blood culture (*n* 24)^b^	
Gram-negative bacilli	
*Bacteroides species*	1
*Brucella species*	1
*Klebsiella pneumoniae*	5
*Serratia marcescens*	1
No growth	16

^a^Pus samples were contributed by 36 out of 37 patients

^b^Blood samples were contributed by 24 out of 37 patients

**Fig. 2:**
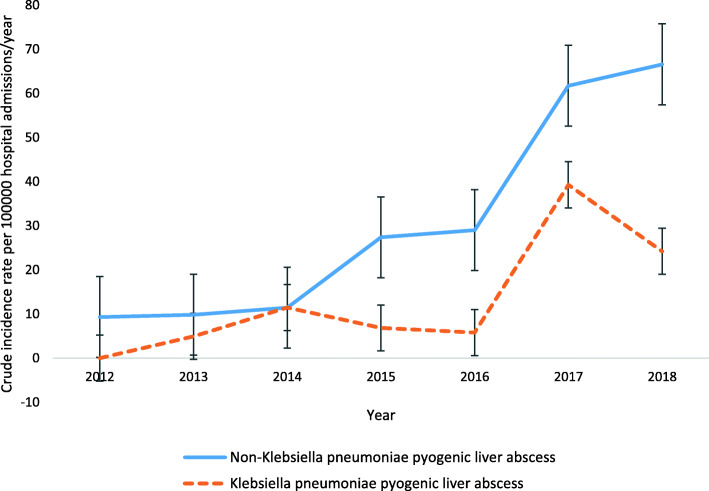
Crude incidence rates per 100,000 hospital admissions/year, with 95% confidence intervals, of *Klebsiella pneumoniae* pyogenic liver abscesses (n 16) and non-*Klebsiella pneumoniae* pyogenic liver abscesses (n 21), Al Ain, United Arab Emirates, 2012–2018

*K. pneumoniae* was the most frequently isolated organism from pus and/or blood cultures of patients with PLA (43.2% [16/37], 95% CI 27.1–60.5%). Furthermore, it was associated with bacteraemia in 31.3% (5/16) of cases. No extra-hepatic complications were observed in *K. pneumoniae* infections. Following logistic regression analysis, no potential risk factors were significantly associated with *K. pneumoniae* infections in patients with PLA (Table 3). Furthermore, the crude annual incidence rates for *K. pneumoniae* were not significantly different from other bacterial pathogens causing PLA during the 7-year period of the study (Fig. 2)*.*

**Table 3:** Potential risk factors associated with *Klebsiella pneumoniae* infection in patients with pyogenic liver abscesses, January 2012 through December 2018, Al Ain, United Arab Emirates

Characteristic	All patients with pyogenic liver abscess	Klebsiella pneumoniae liver abscess	Crude OR^**a**^ (95% CI^**b**^)	Age-adjusted OR (95% CI)	***P*** value^**c**^
Age (years)					
< 40	11	3	1.00		
≥ 40	26	13	2.67 (0.58–12.36)		
Gender					
Female	9	4	1.00		
Male	28	12	0.94 (0.21–4.26)	1.03 (0.22–4.84)	0.971
Region of ethnic origin					
Middle East and North Africa	16	7	1.00		
Indian subcontinent and South East Asia	21	9	0.96 (0.26–3.58)	1.17 (0.30–4.61)	0.825
Health Insurance status					
Insured or self-paying	28	12	1.00		
Free medical assistance	9	4	1.07 (0.23–4.84)	0.84 (0.17–4.05)	0.827
Haemoglobin level (g/L)					
≥ 100	29	12	1.00		
< 100	5	3	2.13 (0.31–14.73)	1.35 (0.18–10.01)	0.769
Total white blood cells					
< 11	11	7	1.00		
≥ 11	23	8	0.30 (0.07–1.36)	0.42 (0.09–2.02)	0.277
Diabetes mellitus					
No	25	9	1.00		
Yes	12	7	2.49 (0.61–10.18)	1.87 (0.39–8.89)	0.433
Malignancy					
No	34	15	1.00		
Yes	3	1	0.63 (0.05–7.67)	0.46 (0.04–5.79)	0.547
Recent abdominal surgery					
No	31	14	1.00		
Yes	6	2	0.6 (0.10–3.82)	0.51 (0.08–3.36)	0.483
Intensive care admission					
No	30	13	1.00		
Yes	7	3	0.98 (0.19–5.17)	0.81 (0.15–4.50)	0.813
Length of hospital stay (days)					
< 10	12	4	1.00		
≥ 10	25	12	1.85 (0.44–7.74)	1.62 (0.37–7.06)	0.524

^a^Odds ratio

^b^Confidence interval

^c^For age-adjusted odds ratio

Overall, extended-spectrum beta-lactamase (ESBL) production was confirmed in 9.4% (3/32) of all Gram-negative bacilli isolated from pus and blood cultures, but none were found to be carbapenemase producers. These ESBL–producing organisms were identified as *Enterobacter cloacae, K. pneumoniae, and Serratia marcescens.*

### Radiological features

Ultrasonography was by far the commonest diagnostic modality used to detect liver abscesses (100%, 45/45). Most liver abscesses were solitary (71.1%, 32/45) and commonly affected the right lobe (73.3%, 33/45). Unilateral pleural effusions were seen on 26.7% (12/45) of chest radiographs. Image-guided percutaneous drainage was used in 87.5% (7/8) of patients with ALA and 100% (37/37) of patients with PLA (Supplementary Table 2). One patient with ALA (1/8, 12.5%) required surgical fenestration and drainage from the outset.

### Treatment outcomes

Table 4 provides a summary of the clinical outcomes of patients with liver abscesses. Overall, 17.8% (8/45) of patients required critical care admission. Most patients were discharged alive (93.3%, 42/45), and 6.7% (3/45) left the hospital against medical advice. No deaths from any cause were recorded within 30 days of hospitalization. Overall, the median length of hospital stay was 12 days (IQR 7–16). Patients with ALA appeared to have shorter hospital stay (7.5 days, IQR 7–8.5) than those with PLA (14 days, IQR 9–17).

**Table 4:** Treatment outcomes of patients with liver abscesses, January 2012 through December 2018, Al Ain, United Arab Emirates

Characteristic	All [*n* 45]	Amoebic liver abscess [*n* 8]	Pyogenic liver abscess [*n* 37]
Intervention in addition to antimicrobial therapy *n* (%)			
Image-guided percutaneous drainage	44 (97.8)	7 (87.5)	37 (100)
Surgical fenestration and drainage	1 (2.2)	1 (12.5)	0 (0.0)
Conservative management	0 (0)	0 (0.0)	0 (0.0)
Clinical outcome *n* (%)			
Critical care admission	8 (17.8)	1 (12.5)	7 (18.9)
Live discharge	42 (93.3)	7 (87.5)	35 (94.6)
Died within 30 days of admission	0 (0)	0 (0)	0 (0)
Left against medical advice	3 (6.7)	1 (12.5)	2 (5.4)
Length of hospital stay [days, median (interquartile range)]	12 (7–16)	7.5 (7–8.5)	14 (9–17)

## Discussion

We reported relatively high crude incidence rates for liver abscesses in our setting: 35.9/100,000 hospital admissions/year and 5.9/100,000 inhabitants/year. A 10-year study from the United Kingdom (UK) reported overall crude annual incidence rates of liver abscesses of 18.2/100,000 hospital admissions and 2.3/100,000 inhabitants [10]. Although there are published case series on liver abscesses from Saudi Arabia, Qatar, and Iraq, none of these studies reported crude annual incidence rates to set regional benchmarks or allow direct comparison with our data [11–13]. Furthermore, we reported an annual incidence rate of 4.9/100,000 inhabitants for PLA. However, it is important to note the significant global variation in PLA annual incidence rate ranging from 17.6/100,000 inhabitants in Taiwan and 3.6/100,000 inhabitants in the United States [2].

The finding that most of our patients with liver abscesses were relatively young at presentation (median age 39 years for ALA and 46 years for PLA) concurs with previous reports from our region [11–13]. However, it contrasts with reports from the Western hemisphere in which older age at presentation was documented [2, 10, 14–16]. Male preponderance was evident in our series (87.5% for ALA, 75.7% for PLA) and is consistent with similar studies from our region [11–13].

A study from Saudi Arabia and the UK found that all patients with ALA were either from or had recently travelled to the Indian subcontinent [11]. Likewise, a study on Israeli travellers found that the vast majority of them had travelled to the Indian subcontinent before the onset of symptoms [17]. Recent data from the United Nations Department of Economic and Social Affairs indicate that migrants comprise a staggering 87.9% of UAE’s total population [18]. Leading countries of origin of these migrants, who are mostly males, are India (39.8%), Bangladesh (12.6%), and Pakistan (11.4%). These facts, together with our observation that ALAs were exclusively found in immigrants from the Indian subcontinent, strongly suggest that ALAs were most likely imported into UAE. Of note, a large study on migrant workers in the Emirate of Sharjah in UAE showed that those from the Indian subcontinent had a significantly higher burden of protozoal and helminths infections [19]. Furthermore, a laboratory-based study by the same research group reported a detection rate of 13.3% of *E. histolytica* in stool samples using molecular methods; however, the authors provided no information as to the ethnic origins of their study participants [20].

Indirect haemagglutination is a reliable method for the serodiagnosis of ALA. A large study from Kuwait evaluated its diagnostic utility for ALA against the ImmunoTab and enzyme-linked immunosorbent assays and found it to have 99% sensitivity and > 95% specificity [21]. An earlier study from Saudi Arabia reported similar validity estimates in patients with ALA [22].

Nearly two-thirds of our PLA cases had a culture-confirmed monomicrobial infection. This is consistent with the UK findings in which 47% of PLAs were due to a single organism but contrasts with data from Spain in which polymicrobial infections predominated [14, 15]. These differences are most likely a reflection of the limitations of traditional culture methods as it has been shown that molecular techniques such as 16 s ribosomal ribonucleic acid testing can increase the diagnostic yield of culture-negative pus samples by 13% [14]. Furthermore, pyrosequencing has been shown to identify an aetiologic pathogen in all patients with abscesses involving the liver, brain, or pleura and that it was significantly more likely than culture to reveal the polymicrobial nature of these infections [23].

With a high prevalence of 43.2%, this study highlights the importance of *K. pneumoniae* as a major pathogen for PLA in our setting. A study from Qatar conducted during 2009–2010 reported a 37.5% prevalence for *K. pneumoniae* among patients with PLA [12]. Another study combining data from Saudi Arabia and the UK during 1995–2005 reported a 23.3% prevalence for *Klebsiella species* in PLA [11]. However, it did not stratify the results by exact species or country of study [11]. In contrast to these relatively recent studies, an earlier study from Iraq conducted in the 1980s showed that *E. coli* was the most commonly isolated organism in patients with PLA, and this observation, when contrasted with recent findings, highlights the change in regional trends since that time [13].

The emergence of the hypervirulent strains of *K. pneumoniae* and their subsequent global spread over the past two decades raises several diagnostic and clinical challenges [24, 25]. For example, phenotypic detection of a hypermucoviscous strain of *K. pneumoniae* entails performing a string test on an agar plate. In contrast, the detection of genetically encoded virulence factors such as K1 or K2 capsular antigens, mucoviscosity-associated gene A (magA), regulator of mucoid phenotype A gene (rmpA), and aerobactin involves complex molecular methods. However, these tests are not routinely performed in clinical diagnostic laboratories in many settings, including ours, mainly because of the prohibitive cost, labour-intensive nature of testing, and limited local expertise. Furthermore, the phenotyping results of *K. pneumoniae* may be of uncertain clinical significance as not all infections with serotypes K1 or K2 translate into an invasive disease [25]. This has been the case in our setting as no extra-hepatic complications were observed despite the dominance of *K. pneumoniae*. Furthermore, only one out of all *K. pneumoniae isolates responsible for PLA in 16 patients* had evidence of ESBL production, and none were carbapenemase producers. This low prevalence of antimicrobial resistance among our *K. pneumoniae* isolates is reassuring but should not leave any room for complacency.

Mucoid *K. pneumoniae* isolates with rmpA and K1 virulence factors were reported in two diabetic patients presenting with PLA in Saudi Arabia – one of them developed endogenous endophthalmitis [26]. Two other *K. pneumoniae* PLA and endogenous endophthalmitis cases were reported in diabetic patients from Saudi Arabia, but no characterization of virulence factors was carried out [27]. Neither of these studies provided detailed information on the antimicrobial susceptibility profile of *K. pneumoniae* isolates.

A large study from mainland China identified invasive *K. pneumoniae* as the leading cause for PLAs and showed that diabetes mellitus, hypertension, and fatty liver disease were the key risk factors [28]. Diabetes mellitus was documented in 32.4% of our PLA patients, 18.8% of PLA patients in Saudi Arabia, and 35.7% in Qatar [11, 12]. Although UAE nationals and non-Arab Asians living in UAE have a similar burden of diabetes mellitus and pre-diabetic state [29], no UAE national was found to have *K. pneumoniae* liver abscess during the 7-year period of our study. This observation, coupled with the fact that a sizeable proportion of *K. pneumoniae* PLA were found in Asians from the Indian subcontinent, calls for further research into possible explanations as to why we UAE nationals, who have a similar prevalence of diabetes mellitus, did not contribute any PLAs to our study.

Ultrasonography was used to detect liver abscesses in all of our patients. Furthermore, plain chest radiography was useful in detecting concomitant unilateral pleural effusions in a quarter of cases in our setting and a fifth of Saudi Arabia cases [11]. These findings confirm the utility of these cheap and widely available diagnostic tools in aiding the diagnosis of liver abscesses. Moreover, image-guided drainage, coupled with appropriate antimicrobial therapy, has been an integral part in managing liver abscesses – only one patient in our series required surgical fenestration.

Data on the response to amoebicidal therapy from our region have been mixed. A 2-year prospective study of 12 ALA patients from Iraq responded very well to metronidazole treatment, and none of them required drainage [30]. Furthermore, a case series from Kuwait on 19 ALA patients with sluggish response to amoebicidal therapy reported rapid clinical recovery and complete abscess resolution following adjunctive percutaneous drainage [31]. In our study, all patients with ALA received amoebicidal therapy coupled with percutaneous or surgical drainage. This practice may be explained in part by previous local experience with severe ALA cases [32]. A large study from India on 144 ALA patients showed that nearly 50% of them failed to respond to amoebicidal therapy and required percutaneous drainage [33]. All these findings highlight the need for better evidence to guide ALA management.

Although we acknowledge the limitations of the retrospective design for this study, we would like to point out that it is the first series from the Arabian Peninsula to report crude annual incidence rates for liver abscesses over a well-defined period. Incomplete or absent data, e.g., travel history and short follow-up duration, are examples of the limitations. Furthermore, the small sample size of patients with PLA did not adequately power the multivariate regression model examining potential risk factors associated with *K. pneumoniae* PLA. Although this study was based at a regional infectious’ disease centre in the Emirate of Abu Dhabi, caution should be exercised when extrapolating our findings to other parts of UAE.

## Conclusions

This 7-year retrospective single-centre cohort study from the UAE provides useful insights into liver abscesses’ epidemiological and clinical features. Furthermore, it estimates their crude annual incidence rates and clinical outcomes and highlights the importation of ALA and predominance of *K. pneumoniae* infections in PLA. Further research is needed to characterize *K. pneumoniae* isolates and better understand the risk factors associated with it in patients presenting with PLA in our setting.

## Supplementary Information

**Additional file 1: Supplementary Table 1.** Laboratory features of patients with liver abscesses, Al Ain, UAE, from January 2012 through December 2018. **Supplementary Table 2.** Radiological features of patients with liver abscesses, Al Ain, UAE, in from January 2012 through December 2018.

## Data Availability

The data underlying this article are presented within the manuscript and the supplementary materials.

## Abbreviations

ALA: Amoebic liver abscess
PLA: Pyogenic liver abscess
UAE: United Arab Emirates
REDCap: Research Electronic Data Capture
MEWS: Modified early warning scores
IQR: Interquartile range
OR: Odds ratio
CI: Confidence interval
magA: Mucoviscosity-associated gene A
rmpA: Regulator of mucoid phenotype A gene
