# Supplementary material for: Importation of Entamoeba histolytica and predominance of Klebsiella pneumoniae in liver abscesses: a 7-year retrospective cohort study from the United Arab Emirates
Source: Trop Dis Travel Med Vaccines. 2021 Jun 12;7:17. doi: 10.1186/s40794-021-00140-8 (PMC8196433; doi:10.1186/s40794-021-00140-8)
Supplement: Supplementary file 1 — Additional file 1: Supplementary Table 1. Laboratory features of patients with liver abscesses, Al Ain, UAE, from January 2012 through December 2018. Supplementary Table 2. Radiological features of patients with liver abscesses, Al Ain, UAE, in from January 2012 through December 2018. [file 40794_2021_140_MOESM1_ESM.docx]

**Supplementary Table 1:** Laboratory features of patients with liver abscesses, Al Ain, UAE, from January 2012 through December 2018

| **Characteristic (**reference range) | **Amoebic liver abscess** | |  | **Pyogenic liver abscess** | |
| --- | --- | --- | --- | --- | --- |
|  | n | median (IQR*) |  | n | median (IQR) |
| Haemoglobin (117–155 g/L) | 8 | 121 (86–131.5) |  | 34 | 116 (111­–130) |
| Total white cell count (4.5–11× 10^9^/L) | 8 | 22.2 (16.6–31.8) |  | 34 | 13.8 (9.1–17.2) |
| Platelets (140–400 × 10^9^/L) | 8 | 368.5 (262.5–496) |  | 34 | 287 (165–363) |
| Prothrombin time (9.5–13.5 second) | 8 | 13.6 (13.3–16.8) |  | 34 | 13 (11.9–14.1) |
| Serum total bilirubin (≤21 μmol/L) | 8 | 23.6 (16.2–34.2) |  | 33 | 27.6 (13.1–38.1) |
| Serum direct bilirubin (≤5 μmol/L) | 8 | 9.1 (6.9–12.2) |  | 34 | 12.8 (4.5–26.3) |
| Serum albumin (35–52 g/L) | 8 | 32 (23.5–33) |  | 31 | 23 (21–28) |
| Serum alkaline phosphatase (40–129 IU/L) | 8 | 173.5 (93–188.5) |  | 34 | 195 (119–387) |
| Serum [alanine transaminase (≤33 IU/L)](https://www.healthline.com/health/alt) | 8 | 52 (27.5–126.2) |  | 34 | 62 (39–126) |
| Serum aspartate transaminase (≤32 IU/L) | 8 | 40.5 (25–90) |  | 32 | 50.5 (36–144) |
| Serum gamma-glutamyl transferase (5–36 IU/L) | 3 | 110 (62–136) |  | 19 | 125 (82–196) |
| Serum urea (2.76–8.07 mmol/L) | 8 | 4.1 (3.4–5.6) |  | 34 | 4.8 (3.7–7.7) |
| Serum creatinine (60–106 μmol/L) | 8 | 84 (73.7–123.8) |  | 34 | 82.3 (72.4–102.6) |
| *Interquartile range |  |  |  |  |  |

**Supplementary Table 2:** Radiological features of patients with liver abscesses, Al Ain, UAE, in from January 2012 through December 2018

| **Characteristic** | **Amoebic liver abscess** | **Pyogenic liver abscess** |
| --- | --- | --- |
| Imaging modality n (%) | [n=8] | [n=37] |
| Ultrasonography | 8 (100) | 37 (100) |
| Computed tomography | 7 (87.5) | 32 (86.5) |
| Magnetic resonance | 0 (0) | 3 (8.1) |
| Abscess type/number n (%) | [n=8] | [n=37] |
| Solitary | 8 (100) | 24(64.9) |
| Multiple | 0 (0.0) | 13 (35.1) |
| Location of abscess n (%) | [n=8] | [n=37] |
| Right lobe | 7 (87.5) | 26 (70.3) |
| Left lobe | 1 (12.5) | 8 (21.6) |
| Both lobes | 0 (0.0) | 3 (8.1) |
| Chest X-ray done n (%) | [n=8] | [n=37] |
| Yes | 6 (75) | 31 (83.8) |
| No | 2 (25) | 6 (16.2) |
| Interpretation of chest x-ray n (%) | [n=6] | [n=31] |
| Normal | 4 (66.7) | 16 (51.6) |
| Abnormal | 2 (33.3) | 15 (48.4) |
| Type of chest X-ray abnormality n (%) | [n=8] | [n=37] |
| Pleural effusion | 1 (12.5) | 11 (29.7) |
| Sub-phrenic collection | 00 (0.0) | 00(0.0) |
| Other | 1 (12.5) | 6 (16.2) |
